# Targeted next generation sequencing of mucosal melanomas identifies frequent *NF1* and *RAS* mutations

**DOI:** 10.18632/oncotarget.16542

**Published:** 2017-03-24

**Authors:** Ioana Cosgarea, Selma Ugurel, Antje Sucker, Elisabeth Livingstone, Lisa Zimmer, Mirjana Ziemer, Jochen Utikal, Peter Mohr, Christiane Pfeiffer, Claudia Pföhler, Uwe Hillen, Susanne Horn, Dirk Schadendorf, Klaus G. Griewank, Alexander Roesch

**Affiliations:** ^1^ Department of Dermatology, University Hospital Essen, West German Cancer Center, University Duisburg-Essen and the German Cancer Consortium (DKTK), University of Duisburg-Essen, Duisburg/Essen, Germany; ^2^ Department of Dermatology, Venereology and Allergology, University Hospital Leipzig, Germany; ^3^ Skin Cancer Unit, German Cancer Research Center (DKFZ), Heidelberg and Department of Dermatology, Venereology and Allergology, University Medical Center Mannheim, Ruprecht-Karl University of Heidelberg, Mannheim, Germany; ^4^ Department of Dermatology, Elbe Klinikum Buxtehude, Buxtehude, Germany; ^5^ Department of Dermatology, Klinikum Augsburg, Augsburg, Germany; ^6^ Department of Dermatology, Saarland University Medical School, Homburg/Saar, Germany

**Keywords:** mucosal melanoma, *NF1*, *RAS*, sequencing, melanoma

## Abstract

**Purpose:**

Mucosal melanoma represents ~1% of all melanomas, frequently having a poor prognosis due to diagnosis at a late stage of disease. Mucosal melanoma differs from cutaneous melanoma not only in terms of poorer clinical outcome but also on the molecular level having e.g. less *BRAF* and more frequent *KIT* mutations than cutaneous melanomas. For the majority of mucosal melanomas oncogenic driver mutations remain unknown.

**Experimental Design and Results:**

In our study, 75 tumor tissues from patients diagnosed with mucosal melanoma were analyzed, applying a targeted next generation sequencing panel covering 29 known recurrently mutated genes in melanoma. *NF1* and *RAS* mutations were identified as the most frequently mutated genes occurring in 18.3% and 16.9% of samples, respectively. Mutations in *BRAF* were identified in 8.4% and *KIT* in 7.0% of tumor samples.

**Conclusions:**

Our study identifies *NF1* as the most frequently occurring driver mutation in mucosal melanoma. *RAS* alterations, consisting of *NRAS* and *KRAS* mutations, were the second most frequent mutation type. *BRAF* and *KIT* mutations were rare with frequencies below 10% each. Our data indicate that in mucosal melanomas *RAS*/*NF1* alterations are frequent, implying a significant pathogenetic role for MAPK and potentially PI3K pathway activation in these tumors.

## INTRODUCTION

Mucosal melanomas arise from melanocytes of the mucosal membrane and represent a rare subgroup of melanoma, accounting for around 1% of all melanomas [1]. Frequently diagnosed at an advanced tumor stage, they generally have a poor prognosis [2]. In contrast to cutaneous melanomas, where exogenous or endogenous risk factors such as UV-exposure or genetic predisposition are well known and studied in a high number of patients [3], no comparable factors have been identified for mucosal melanomas. Additionally, little information is available with regard to the molecular pathogenesis of mucosal melanoma. In general, mucosal melanomas seem to have a lower overall mutational burden as compared to cutaneous melanomas (8193 vs. 86495 somatic single nucleotide variants per tumor) [4, 5] but a higher number of chromosomal aberrations [6]. However, *BRAF* V600 mutations, the most frequent and therapeutically best-targetable gene alteration in cutaneous melanoma (present in ~50% of cases) is only rarely found in mucosal melanomas (≤ 10% of cases), which limits clinical treatment options [6–8]. Other activating oncogenic events, e.g. gene amplifications or gain-of-function mutations of *KIT* are more frequently detected in mucosal melanomas. Existing literature reports *KIT* activation in 15-39% of mucosal melanomas [9–13]. However, *KIT*-targeting therapies, e.g. with imatinib, have failed to show convincing therapeutic efficiency in mucosal melanoma in larger studies [14, 15]. *NRAS* mutations are somewhat less frequent in mucosal (10-20%) [6, 8] than cutaneous melanomas (20-30%) [6, 16–18]. Activating mutations in *GNAQ* and *GNA11*, which are commonly detected in uveal melanoma [19], have recently been reported to occur in 9.5% of mucosal melanomas [20], a finding not reported in previous studies [21]. Many existing studies have been performed on cohorts with limited sample numbers.

In summary, mucosal melanoma represents a clinically aggressive cancer entity rarely harboring known therapeutically targetable driver mutations. Our study aimed to identify additional oncogenic driver mutations in mucosal melanoma in a larger cohort of patients to recognize additional molecular pathways with the potential to be exploited for establishing future therapeutic strategies.

## RESULTS

### Patient characteristics

Samples were obtained from 75 patients, 46 females and 29 males. Clinic-pathological characteristics are summarized in Table 1. In 4 cases, sequencing analysis was not possible due to poor sequencing quality; clinico-pathological characteristics of these patients are not included in Table 1.

**Table 1:** Clinicopathological characteristics of the patients

Variable	Total (n=71)	*NF1*	WT	p-value	*RAS*	WT	p-value	*BRAF*	WT	p-value
**Median age (range)**	64 (33-84)									
**Gender**										
Male	26	3	23	0.348	8	18	0.024	2	24	1.0
Female	45	10	35		4	41		4	41	
**Anatomical site**										
Head and neck	28	3	25	0.386	9	19	0.224	2	26	0.672
Genital area	25	5	20		3	22		2	23	
Anorectum	9	3	6		0	9		1	8	
Digestive tract	3	1	2		0	3		1	2	
Urinary tract	3	0	3		0	3		0	3	
Data missing	3	1	2		0	3		0	3	
**Sample type**										
Primary tumor	41	8	33	0.798	8	33	0.936	5	36	0.837
Metastasis	22	5	17		3	19		1	21	
Recurrence	3	0	3		0	3		0	3	
Data missing	5	0	5		1	4		0	5	

### Targeted next generation sequencing

**Figure 1:**
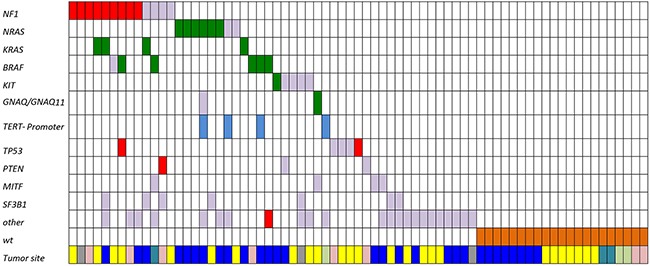
Mutation distribution in mucosal melanomas Green: mutations known or assumed to be activating; red: loss of function mutations; blue: mutations in the *TERT* promoter region; grey: missense mutation (frequently with unknown functional consequences); brown: wild-type samples (showing no mutation in the analyzed gene panel). Tumor location: Yellow, genital area; light pink, anorectum; dark blue, head and neck; light green, urinary tract; petrol, digestive tract; grey, data missing.

**Figure 2:**
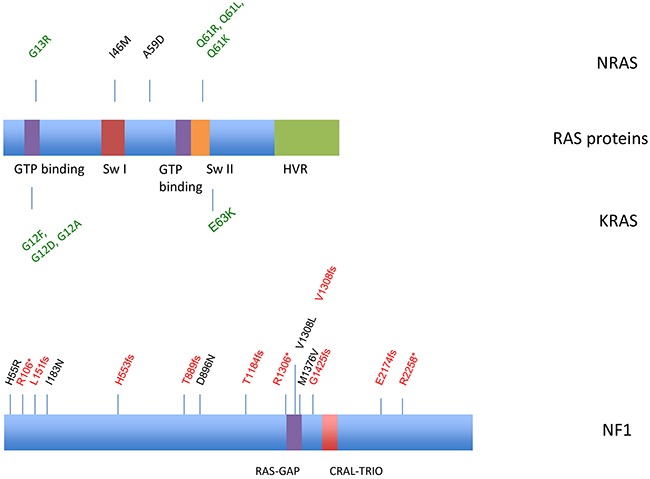
Distribution of identified *NRAS, KRAS* and *NF1* mutations Frameshift and nonsense mutations are annotated in red, activating mutations are demonstrated in green. Missense mutations are black. Switch I, effector/GAP interaction; Switch II, EF interaction; HVR, hypervariable region. NF1 contains a Ras-GAP domain (GTPase-activator protein for Ras-like GTPase) and a CRAL-TRIO domain.

Mutations were identified in 50 samples (Figure 1, Table 2, Supplementary Table 1). *NF1* and *RAS* were the most frequently mutated genes (Figure 2, Supplementary Figure 1 and 2, Table 2, Supplementary Table 1), with 15 *NF1* mutations identified in 13 samples (18.3.%) and 12 *RAS* mutations identified in 12 samples (16.9%). In 9 out of 13 samples (69.2%) a clearly inactivating *NF1* mutation was present resulting in non-sense (synthesis stop) or frameshift mutations. Examples of some inactivating *NF1* mutations detected in our cohort are shown in Supplementary Figure 1. Two samples harbored multiple *NF1* mutations (Table 2, Supplementary Table 1); one of them having two inactivating mutations, the other one having an inactivating and a D896N missense mutation. *RAS* gene alterations were found in 12 out of 71 samples (16.9%), including 8 *NRAS* and 4 *KRAS* mutations. Sanger sequencing was performed to validate the identified *KRAS* mutations (Supplementary Figure 3). Six samples (8.4%) harbored *BRAF* mutations, 5 of which were activating V600 mutations (4 V600E and 1 V600K) and 1 N188S mutation (Supplementary Table 2). The mutation pattern on protein level for the identified *NRAS, KRAS* and *NF1* mutations are shown in Figure 2. *KIT* mutations were detected in 5 samples (7%), but only 1 was a known activating mutation. Another 4 samples carried known activating *TERT*-promoter mutations. One *GNA11* S267F mutation and 1 *GNAQ* R183Q mutation were identified. Examples of the most frequent activating mutations identified are illustrated in Supplementary Figure 2. Three samples harboring *KRAS* mutations also had concurrent *NF1* mutations, 2 of which were clearly functionally inactivating. Additionally, 5 *TP53*, 7 *SF3B1*, 4 *MITF* and 3 *PTEN* mutations were identified. Other less frequent mutations were identified in various genes including *SMARC, BAP1, TERT, WT1, PIK3CA, MAP2K2, CDK4, CTNNB1, RAC1, ARID2 and ARID1A*. In 21 samples no non-synonymous protein coding mutation were identified in the 29 genes analyzed.

**Table 2:** List of identified mutations

Nr.	Type	Location primary	NF1	RAS	BRAF	Other Mutations
**1**	**M**	**G**	**E2174fs; L151fs**			
**2**	**M**	**DM**	R106*			
**3**	**M**	**G**	R2258*	**KRAS G12D**		
**4**	**P**	**A**	**V1308fs**			**TERT S663N**
**5**	**P**	**G**	**G1425fs**		**V600E**	TP53 Q165*
**6**	**P**	**HN**	**H553fs**			**PIK3CA E109del**
**7**	**P**	**G**	**T889fs**		**N188S**	
**8**	**M**	**A**	R1306*			
**9**	**P**	**HN**	**T1184fs; D896N**	**KRAS G12A**		**ARID1A V700A; SF3B1 D894N**
**10**	**M**	**G**	**H55R**			
**11**	**P**	**A**	**I183N**			**PTEN K163fs; SF3B1 R625H**
**12**	**P**	**D**	**M1376V**		**V600E**	**ARID1A R1202Q; ARID2 T1208A; MITF A401S**
**13**	**P**	**HN**	**V1308L**	**KRAS E63K**		**SF3B1 R625H**
**14**	**P**	**HN**		**KRAS G12F**		
**15**	**P**	**HN**		**NRAS Q61R**		
**16**	**P**	**HN**		**NRAS Q61R**		
**17**	**P**	**HN**		**NRAS Q61K**		
**18**	**P**	**HN**		**NRAS G13R**		**TERT Ser1104Thr**
**19**	**P**	**HN**		**NRAS Q61K**		**TERT P C228T; RAC1 N92K; GNA11 S267F;**
**20**	**U**	**G**		**NRAS Q61L**		**SF3B1 V634A**
**21**	**M**	**HN**		**NRAS A59D**		**TERT P C243T; TERT P C252T; SMARCA4 A152T**
**22**	**M**	**V**		**NRAS I46M**		
**23**	**M**	**HN**			**V600E**	**TERT P C250T**
**24**	**P**	**HN**			**V600E**	**PIK3CA L896fs**
**25**	**P**	**A**			**V600K**	
**26**	**M**	**HN**				**KIT L576P**
**27**	**M**	**Ur**				**TERT P C228T; WT1 D497N**
**28**	**M**	**G**				**GNAQ R183Q; MITF V487I**
**29**	**P**	**G**				**TP53 P58fs**
**30**	**P**	**G**				**ARID2 Y612C**
**31**	**M**	**HN**				**TERT L1002V**
**32**	**M**	**A**				**PTEN L108R**
**33**	**M**	**G**				**PIK3R1 T239M**
**34**	**P**	**G**				**KIT Y553del; MAP2K2 G286R**
**35**	**P**	**G**				**TERT R819H**
**36**	**P**	**HN**				**MITF N267K**
**37**	**P**	**A**				**TP53 C135R**
**38**	**R**	**HN**				**KIT V50L; PTEN C136R**
**39**	**P**	**G**				**TP53 P151A**
**40**	**U**	**G**				**ARID2 M545I; SF3B1 R625H**
**41**	**P**	**G**				**TERT R819H**
**42**	**U**	**A**				**MITF V487I; SMARCA4 R1260S**
**43**	**R**	**HN**				**SF3B1 T916S; CK4 R209C**
**44**	**P**	**HN**				**TP53 R175G**
**45**	**P**	**G**				**KIT, L783I**
**46**	**M**	**DM**				**KIT, I748T; BAP1, G579R: SF3B1, R625L**
**47**	**P**	**HN**				**BAP1 Y646C**
**48**	**P**	**HN**				**CTNNB1 Y331C**
**49**	**P**	**HN**				**TERT S953F**
**50**	**M**	**DM**				**CK4 V174M**

Green, mutations known to be activating; red, loss of function mutations; black, missense mutation with unknown functional consequences. Abbreviations: *Nr*. sample number; *M* metastasis; *P* primary tumor; *R recurrence*; *U* unknown; *fs* frame shift; * = stop codon (nonsense mutation); *HN* Head and Neck, *G* Genital area, *A* Anorectum, *D* Digestive tract, *Ur* Urinary tract, *DM* data missing.

For more details including allele frequencies and cDNA annotations, see Supplementary Table 1.

### Statistical analysis

We performed a statistical analysis to assess possible associations of clinical parameters such as gender, location of the primary tumor and sample type (primary, metastasis or recurrence) with the *NF1*, *RAS* and *RAF* mutational status. A statistically significant association was determined between *RAS* mutational status and male sex (p=0.024, Table 1).

## DISCUSSION

In this study, 71 mucosal melanoma samples were screened for mutations in known recurrently mutated genes in cutaneous and uveal melanoma. The most frequent mutations were identified in the *NF1* gene and *RAS* gene family members, indicating that mucosal melanomas have a genetic mutation profile which is different from that of cutaneous or uveal melanomas. Our study identified an unexpectedly high number of *NF1* mutations. In 13 (18.3,%) out of 71 samples, *NF1* mutations were identified. Nine of those (69.2%) harbored clearly inactivating mutations, i.e. nonsense or frameshift mutations.

To our knowledge, there is no previous data demonstrating that mucosal melanomas express such a high frequency of *NF1* mutations. Yang *et al*. recently conducted a targeted sequencing analysis on 15 anorectal melanomas and identified that 3 of these tumors harbored an *NF1* mutation [22]. In recent years, *NF1* has been recognized as the third most commonly mutated gene (after *BRAF* and *RAS*) resulting in activation of the MAPK pathway with a reported mutation frequency of 14% [18]. In cutaneous melanomas, more than half of the mutations reported are loss-of-function (LoF) events. In the mucosal melanomas studied here, 9 out of 13 cases carried *NF1* LoF mutations (69.2%). It is known that *NF1* is a GTPase-activating protein which downregulates the activity of the RAS protein [18]. As such, LoF mutations in *NF1* are an important genetic mechanism for constitutive MAPK pathway activation.

A relevant role for *NF1* mutations in cutaneous melanomas lacking conventional (i.e. *BRAF* or *NRAS*) activating mutations has been already highlighted by other studies. Wiesner *et al*. demonstrated that desmoplastic melanomas frequently harbor *NF1* mutations [23]. Desmoplastic melanomas are typically associated with high UV-exposure and high mutational loads. Congruently, Krauthammer *et al*. described *NF1* mutations in association with mutations in RASopathy genes (e.g. *RASA2*, *PTPN11*, etc.) in cutaneous melanomas with evidence of high sun-exposure [24]. The association of UV-exposure with *NF1* mutations observed in cutaneous melanoma is not to be expected in mucosal melanoma [4, 5]. Although mutational mechanisms may differ, these studies and our data support *NF1* mutations being highly relevant in melanoma subgroups that rarely harbor *BRAF* or *NRAS* mutations.

Twelve out of 71 (16.9%) mucosal melanomas analyzed harbored *RAS* mutations, 8 in the *NRAS* and 4 in the *KRAS* gene. No mutations were identified in *HRAS*. Previous studies have focused primarily on *NRAS*, where the mutation frequency ranges from 5% to 15% [6, 8, 25–27]. The frequency of *NRAS* mutations detected in our study with 11.2% is comparable. *KRAS* mutations have not been assessed in most previous studies of mucosal melanoma, however, accounted for one third of the mutations in *RAS* genes observed in our cohort. A slightly significant association of *RAS* mutation status with male gender was noted (p=0.024). This potential association will need be to assessed in future studies with considerably larger sample numbers.

*BRAF* was the third most frequently mutated gene identified in our study. Of the 6 mutations (8.4%) identified, 5 were well known V600 activating mutations, consisting of 4 V600E and 1 V600K mutation. The other identified mutation, resulting in an N188S exchange, is of unclear significance and could be a non-relevant passenger mutation. These findings are in accordance with reports stating that activating *BRAF* mutations in mucosal melanomas are rare with a frequency between 5 and 17% [8, 9, 26, 28, 29]. Only one patient in our cohort received BRAF inhibitor therapy showing a partial response (Supplementary Table 2). The efficacy of BRAF inhibitor therapies in mucosal melanomas will need to be further assessed in larger studies.

Genetic alterations of *KIT*, including mutations and copy number increases, have been reported to occur in up to 39% of mucosal melanomas [9–13]. In our study, 5 out of 71 (7.0%) samples had a *KIT* mutation; 2 mutations in tumors of the head and neck region and 2 in vulvar melanomas. Omholt et al. [27] reported *KIT* mutations in 17% (n=12) of primary mucosal melanomas. They found that 35% (8 out of 23) of vulvar melanomas harbored *KIT* mutations. In our study, 2 out of 9 (22.2%) vulvar melanomas had a *KIT* mutation. Generally, our findings support existing literature stating that *KIT* mutations are more frequent in melanomas of the genital area followed by melanomas of the head and neck and the anorectal area [8, 26, 30, 31].

*TERT* promoter mutations resulting in increased transcription of the *TERT* gene have been identified as the most common mutation in cutaneous melanoma [32–34]. In congruence with previous studies reporting low mutation rates in mucosal melanomas [35, 36], the mutation frequency in our cohort was 5.6%. All *TERT* mutations were C>T mutations. As C>T alterations are classically associated with UV-exposure [32, 37, 38], the lower *TERT* mutation frequency may be due to the very limited UV-exposure of tumors arising in mucosal locations.

Two alterations were identified in *GNAQ* and *GNA11*, mutations that are usually associated with uveal melanomas and blue nevi [19]. Of those, only the *GNAQ* R183Q mutation is known to be functionally activating, resulting in increased mitogenic signaling in melanocytic tumors and rare vascular diseases such as Sturge-Weber syndrome and phakomatosis pigmentovascularis [39, 40]. The other mutation identified (*GNA11* S267F) is not known to be functionally relevant and probably represents a bystander mutation considering the sample also harbored a *NRAS* Q61K hot-spot mutation. While *GNAQ* and *GNA11* mutations were recently reported to occur in 9.5% of mucosal melanomas [20], our study suggests these mutations are less frequent in this tumor entity.

In out cohort, 31 samples presented mutations in the MAPK pathway (some of them harboring more than one mutation): 13 *NF1*, 12 *RAS*, 6 *RAF*, 5 *KIT*, 1 *GNAQ* and 1 *GNA11* mutation. In 23 of those samples mutations resulting in MAPK activation were found suggesting that this is a critical event in the pathogenesis of mucosal melanoma. This is similar to cutaneous melanoma, where constitutive activation of the MAPK pathway is a known critical event. The genetic alterations leading to this activation however vary between these melanoma subtypes [18].

Although our study represents the most comprehensive genetic analysis of 75 mucosal melanomas presented to date, it does have some limitations. Mutations occurring in genes not covered by our panel could not be identified. Additionally, our approach did not allow us to reliably detect copy number variations.

Our results underline that mucosal melanomas are genetically distinct from cutaneous and uveal melanomas with frequent inactivating mutations in *NF1* and activating mutations in *RAS* genes. Our findings suggest that similar to cutaneous melanoma, activation of the MAPK pathway is a pivotal event in mucosal melanoma. Taken into consideration that both *NF1* and *RAS* alterations can additionally activate PI3K signaling, this pathway could be of particular significance in mucosal melanomas. It stands to reason that other, so far unidentified, mutations are present in mucosal melanomas and future whole-exome or whole-genome studies of larger tumor cohorts will be required to fully elucidate the landscape of genetic alterations involved.

## MATERIALS AND METHODS

### Sample selection

Samples of mucosal melanomas were retrieved from the biobank of the Department of Dermatology, Essen, Germany, from the TRIM (Tissue Registry in Melanoma) project of the German DeCOG (Dermatological Cooperative Oncology Group) as well as from patients enrolled into the DeCOG trial ChemoSensMM [ClinicalTrials.gov: NCT00779714 [41]. Samples were considered mucosal melanoma if the tumor originated in a mucosal site, was diagnosed as melanoma histopathologically (confirmed by at least one immunohistochemical marker [Melan-A, HMB-45 or S100]) and no other preexisting, e.g. cutaneous, melanoma was reported (to exclude potential mucosal metastasis). The study was performed with informed patient consent in accordance with the guidelines of the Ethics Committee of the Medical Faculty of the University Duisburg-Essen (ethical approval no. 15-6473-BO, no. 15-6566-BO).

### DNA isolation

FFPE tissue was prepared in 10 μm sections and deparaffinized according to standard procedures. In brief, 2 steps of 5 min xylene, 5 min 100% ethanol, 5 min 95% ethanol, 5 in 70% ethanol, 5 min 50% ethanol, rinsing in water. After air drying, the tumor tissue was manually macrodissected from the sections. The tumor content in the area of macrodissection was required to be at least 25%, however, was generally considerably higher (documented values are listed in Supplementary Table 1). Genomic DNA was isolated applying the QIAamp DNA Mini Kit (Qiagen, Hilden, Germany) according to the manufacturer's instructions.

### Targeted sequencing

A custom designed amplicon-based sequencing panel covering the *TERT* promoter and the complete coding regions of 29 known recurrently mutated genes in cutaneous and uveal melanoma (Supplementary Table 3) was applied. This panel was initially clinically validated over a period of 3 months, where Sanger and NGS panel sequencing were performed in parallel for all samples analyzed. All known recurrent mutations in melanoma (incl. *BRAF* V600, *NRAS* G12, G13 and Q61 and *KIT* L576, K642 and N822) were repeatedly picked up by our NGS panel which showed a 100% concordance with mutations identified by Sanger sequencing, however demonstrated a higher level of sensitivity. After successful validation, our routine clinical sequencing effort has solely relied on the NGS panel, which in over 2 years has been applied to more than 1300 melanocytic tumors. Our panel sequencing approach is not able to reliably detect copy number variations.

Adapter ligation and barcoding of individual samples occurred applying the NEBNext Ultra DNA Library Prep Mastermix Set and NEBNext Multiplex Oligos for Illumina from New England Biolabs. Sequencing analysis was performed using CLC Cancer Research Workbench from QIAGEN as previously described [42]. In brief, analysis included the following steps: The CLC workflow included adapter trimming and read pair merging before mapping to the human reference genome (hg19). Insertions and deletions as well as single nucleotide variant detection, local realignment and primer trimming followed. Additional information was then obtained regarding potential mutation type, known single nucleotide polymorphisms and conservation scores by cross-referencing various databases (COSMIC, ClinVar, dbSNP, 1000 Genomes Project, HAPMAP and PhastCons_Conservation_scores_hg19). The CLC generated csv files were further analyzed manually with mutations affecting the protein-coding portion of the gene considered if predicted to result in non-synonymous amino acid changes. The average fold coverage was 2585x. To eliminate questionable low frequency background mutation calls, not uncommon in our experience with FFPE amplicon sequencing approaches [43], mutations were reported if overall coverage of the mutation site was ≥30 reads, ≥10 reads reported the mutated variant and the frequency of mutated reads was ≥10 %.

### Statistical analysis

The associations of mutation status with clinical parameters such as gender, location of the primary tumor and sample origin (primary, metastasis or recurrence), was investigated using chi-squared tests and Fisher exact tests as appropriate. Statistical analyses were performed using SPSS 23.0 (IBM Corp., Armonk NY, USA), considering a p-value of p≤0.05 as statistically significant.

## SUPPLEMENTARY MATERIALS FIGURES AND TABLES





## References

[1] Chang AE, Karnell LH, Menck HR, The American College of Surgeons Commission on Cancer and the American Cancer Society (1998). The National Cancer Data Base report on cutaneous and noncutaneous melanoma: a summary of 84,836 cases from the past decade. Cancer.

[2] Manolidis S, Donald PJ (1997). Malignant mucosal melanoma of the head and neck: review of the literature and report of 14 patients. Cancer.

[3] Postow MA, Hamid O, Carvajal RD (2012). Mucosal melanoma: pathogenesis, clinical behavior, and management. Curr Oncol Rep.

[4] Furney SJ, Turajlic S, Stamp G, Nohadani M, Carlisle A, Thomas JM, Hayes A, Strauss D, Gore M, van den Oord J, Larkin J, Marais R (2013). Genome sequencing of mucosal melanomas reveals that they are driven by distinct mechanisms from cutaneous melanoma. J Pathol.

[5] Berger MF, Hodis E, Heffernan TP, Deribe YL, Lawrence MS, Protopopov A, Ivanova E, Watson IR, Nickerson E, Ghosh P, Zhang H, Zeid R, Ren X (2012). Melanoma genome sequencing reveals frequent PREX2 mutations. Nature.

[6] Curtin JA, Fridlyand J, Kageshita T, Patel HN, Busam KJ, Kutzner H, Cho KH, Aiba S, Bröcker EB, LeBoit PE, Pinkel D, Bastian BC (2005). Distinct sets of genetic alterations in melanoma. N Engl J Med.

[7] Davies H, Bignell GR, Cox C, Stephens P, Edkins S, Clegg S, Teague J, Woffendin H, Garnett MJ, Bottomley W, Davis N, Dicks E, Ewing R (2002). Mutations of the BRAF gene in human cancer. Nature.

[8] Tacastacas JD, Bray J, Cohen YK, Arbesman J, Kim J, Koon HB, Honda K, Cooper KD, Gerstenblith MR (2014). Update on primary mucosal melanoma. J Am Acad Dermatol.

[9] Curtin JA, Busam K, Pinkel D, Bastian BC (2006). Somatic activation of KIT in distinct subtypes of melanoma. J Clin Oncol.

[10] Rivera RS, Nagatsuka H, Gunduz M, Cengiz B, Gunduz E, Siar CH, Tsujigiwa H, Tamamura R, Han KN, Nagai N (2008). C-kit protein expression correlated with activating mutations in KIT gene in oral mucosal melanoma. Virchows Archiv.

[11] Antonescu CR, Busam KJ, Francone TD, Wong GC, Guo T, Agaram NP, Besmer P, Jungbluth A, Gimbel M, Chen CT, Veach D, Clarkson BD, Paty PB, Weiser MR (2007). L576P KIT mutation in anal melanomas correlates with KIT protein expression and is sensitive to specific kinase inhibition. International Journal of Cancer.

[12] Ashida A, Takata M, Murata H, Kido K, Saida T (2009). Pathological activation of KIT in metastatic tumors of acral and mucosal melanomas. International journal of cancer.

[13] Carvajal RD, Antonescu CR, Wolchok JD, Chapman PB, Roman RA, Teitcher J, Panageas KS, Busam KJ, Chmielowski B, Lutzky J, Pavlick AC, Fusco A, Cane L (2011). KIT as a therapeutic target in metastatic melanoma. JAMA.

[14] Kim KB, Alrwas A (2014). Treatment of KIT-mutated metastatic mucosal melanoma. Chin Clin Oncol.

[15] Carvajal RD (2013). Another option in our KIT of effective therapies for advanced melanoma. J Clin Oncol.

[16] Hodis E, Watson IR, Kryukov GV, Arold ST, Imielinski M, Theurillat JP, Nickerson E, Auclair D, Li L, Place C, Dicara D, Ramos AH, Lawrence MS (2012). A landscape of driver mutations in melanoma. Cell.

[17] Krauthammer M, Kong Y, Ha BH, Evans P, Bacchiocchi A, McCusker JP, Cheng E, Davis MJ, Goh G, Choi M, Ariyan S, Narayan D, Dutton-Regester K (2012). Exome sequencing identifies recurrent somatic RAC1 mutations in melanoma. Nat Genet.

[18] Cancer Genome Atlas N, Cancer Genome Atlas Network (2015). Genomic Classification of Cutaneous Melanoma. Cell.

[19] Van Raamsdonk CD, Griewank KG, Crosby MB, Garrido MC, Vemula S, Wiesner T, Obenauf AC, Wackernagel W, Green G, Bouvier N, Sozen MM, Baimukanova G, Roy R (2010). Mutations in GNA11 in uveal melanoma. N Engl J Med.

[20] Sheng X, Kong Y, Li Y, Zhang Q, Si L, Cui C, Chi Z, Tang B, Mao L, Lian B, Wang X, Yan X, Li S (2016). GNAQ and GNA11 mutations occur in 9.5% of mucosal melanoma and are associated with poor prognosis. Eur J Cancer.

[21] Pappa KI, Vlachos GD, Roubelakis M, Vlachos DE, Kalafati TG, Loutradis D, Anagnou NP (2015). Low mutational burden of eight genes involved in the MAPK/ERK, PI3K/AKT, and GNAQ/11 pathways in female genital tract primary melanomas. Biomed Res Int.

[22] Yang HM, Hsiao SJ, Schaeffer DF, Lai C, Remotti HE, Horst D, Mansukhani MM, Horst BA (2017). Identification of recurrent mutational events in anorectal melanoma. Mod Pathol.

[23] Wiesner T, Kiuru M, Scott SN, Arcila M, Halpern AC, Hollmann T, Berger MF, Busam KJ (2015). NF1 Mutations Are Common in Desmoplastic Melanoma. Am J Surg Pathol.

[24] Krauthammer M, Kong Y, Bacchiocchi A, Evans P, Pornputtapong N, Wu C, McCusker JP, Ma S, Cheng E, Straub R, Serin M, Bosenberg M, Ariyan S (2015). Exome sequencing identifies recurrent mutations in NF1 and RASopathy genes in sun-exposed melanomas. Nat Genet.

[25] Minor DR, Kashani-Sabet M, Garrido M, O’Day SJ, Hamid O, Bastian BC (2012). Sunitinib therapy for melanoma patients with KIT mutations. Clin Cancer Res.

[26] Omholt K, Grafstrom E, Kanter-Lewensohn L, Hansson J, Ragnarsson-Olding BK (2011). KIT pathway alterations in mucosal melanomas of the vulva and other sites. Clin Cancer Res.

[27] Zebary A, Jangard M, Omholt K, Ragnarsson-Olding B, Hansson J (2013). KIT, NRAS and BRAF mutations in sinonasal mucosal melanoma: a study of 56 cases. Br J Cancer.

[28] Maldonado JL, Fridlyand J, Patel H, Jain AN, Busam K, Kageshita T, Ono T, Albertson DG, Pinkel D, Bastian BC (2003). Determinants of BRAF mutations in primary melanomas. J Natl Cancer Inst.

[29] Turri-Zanoni M, Medicina D, Lombardi D, Ungari M, Balzarini P, Rossini C, Pellegrini W, Battaglia P, Capella C, Castelnuovo P, Palmedo G, Facchetti F, Kutzner H (2013). Sinonasal mucosal melanoma: molecular profile and therapeutic implications from a series of 32 cases. Head Neck.

[30] Satzger I, Schaefer T, Kuettler U, Broecker V, Voelker B, Ostertag H, Kapp A, Gutzmer R (2008). Analysis of c-KIT expression and KIT gene mutation in human mucosal melanomas. Br J Cancer.

[31] Beadling C, Jacobson-Dunlop E, Hodi FS, Le C, Warrick A, Patterson J, Town A, Harlow A, Cruz F, Azar S, Rubin BP, Muller S, West R (2008). KIT gene mutations and copy number in melanoma subtypes. Clin Cancer Res.

[32] Horn S, Figl A, Rachakonda PS, Fischer C, Sucker A, Gast A, Kadel S, Moll I, Nagore E, Hemminki K, Schadendorf D, Kumar R (2013). TERT promoter mutations in familial and sporadic melanoma. Science.

[33] Huang FW, Hodis E, Xu MJ, Kryukov GV, Chin L, Garraway LA (2013). Highly recurrent TERT promoter mutations in human melanoma. Science.

[34] Ekedahl H, Lauss M, Olsson H, Griewank KG, Schadendorf D, Ingvar C, Jönsson G (2016). High TERT promoter mutation frequency in non-acral cutaneous metastatic melanoma. Pigment Cell Melanoma Res.

[35] Griewank KG, Murali R, Puig-Butille JA, Schilling B, Livingstone E, Potrony M, Carrera C, Schimming T, Möller I, Schwamborn M, Sucker A, Hillen U, Badenas C (2014). TERT promoter mutation status as an independent prognostic factor in cutaneous melanoma. J Natl Cancer Inst.

[36] Jangard M, Zebary A, Ragnarsson-Olding B, Hansson J (2015). TERT promoter mutations in sinonasal malignant melanoma: a study of 49 cases. Melanoma Res.

[37] Brash DE, Rudolph JA, Simon JA, Lin A, McKenna GJ, Baden HP, Halperin AJ, Pontén J (1991). A role for sunlight in skin cancer: UV-induced p53 mutations in squamous cell carcinoma. Proc Natl Acad Sci USA.

[38] Lawrence MS, Stojanov P, Polak P, Kryukov GV, Cibulskis K, Sivachenko A, Carter SL, Stewart C, Mermel CH, Roberts SA, Kiezun A, Hammerman PS, McKenna A (2013). Mutational heterogeneity in cancer and the search for new cancer-associated genes. Nature.

[39] Thomas AC, Zeng Z, Rivière JB, O’Shaughnessy R, Al-Olabi L, St-Onge J, Atherton DJ, Aubert H, Bagazgoitia L, Barbarot S, Bourrat E, Chiaverini C, Chong WK (2016). Mosaic Activating Mutations in GNA11 and GNAQ Are Associated with Phakomatosis Pigmentovascularis and Extensive Dermal Melanocytosis. J Invest Dermatol.

[40] Shirley MD, Tang H, Gallione CJ, Baugher JD, Frelin LP, Cohen B, North PE, Marchuk DA, Comi AM, Pevsner J (2013). Sturge-Weber syndrome and port-wine stains caused by somatic mutation in GNAQ. N Engl J Med.

[41] Ugurel S, Schadendorf D, Pföhler C, Neuber K, Thoelke A, Ulrich J, Hauschild A, Spieth K, Kaatz M, Rittgen W, Delorme S, Tilgen W, Reinhold U, Dermatologic Cooperative Oncology Group (2006). *In vitro* drug sensitivity predicts response and survival after individualized sensitivity-directed chemotherapy in metastatic melanoma: a multicenter phase II trial of the Dermatologic Cooperative Oncology Group. Clin Cancer Res.

[42] van de Nes J, Gessi M, Sucker A, Möller I, Stiller M, Horn S, Scholz SL, Pischler C, Stadtler N, Schilling B, Zimmer L, Hillen U, Scolyer RA (2016). Targeted next generation sequencing reveals unique mutation profile of primary melanocytic tumors of the central nervous system. J Neurooncol.

[43] Murali R, Chandramohan R, Möller I, Scholz SL, Berger M, Huberman K, Viale A, Pirun M, Socci ND, Bouvier N, Bauer S, Artl M, Schilling B (2015). Targeted massively parallel sequencing of angiosarcomas reveals frequent activation of the mitogen activated protein kinase pathway. Oncotarget.

