# Supplementary material for: Targeted next generation sequencing of mucosal melanomas identifies frequent NF1 and RAS mutations
Source: Oncotarget. 2017 Mar 24;8(25):40683–92. doi: 10.18632/oncotarget.16542 (PMC5522195; doi:10.18632/oncotarget.16542)
Supplement: Supplementary file 1 [file oncotarget-08-40683-s001.pdf]

Targeted next generation sequencing of mucosal melanomas identifies frequent *NF1* and *RAS* mutations

SUPPLEMENTARY FIGURES AND TABLES

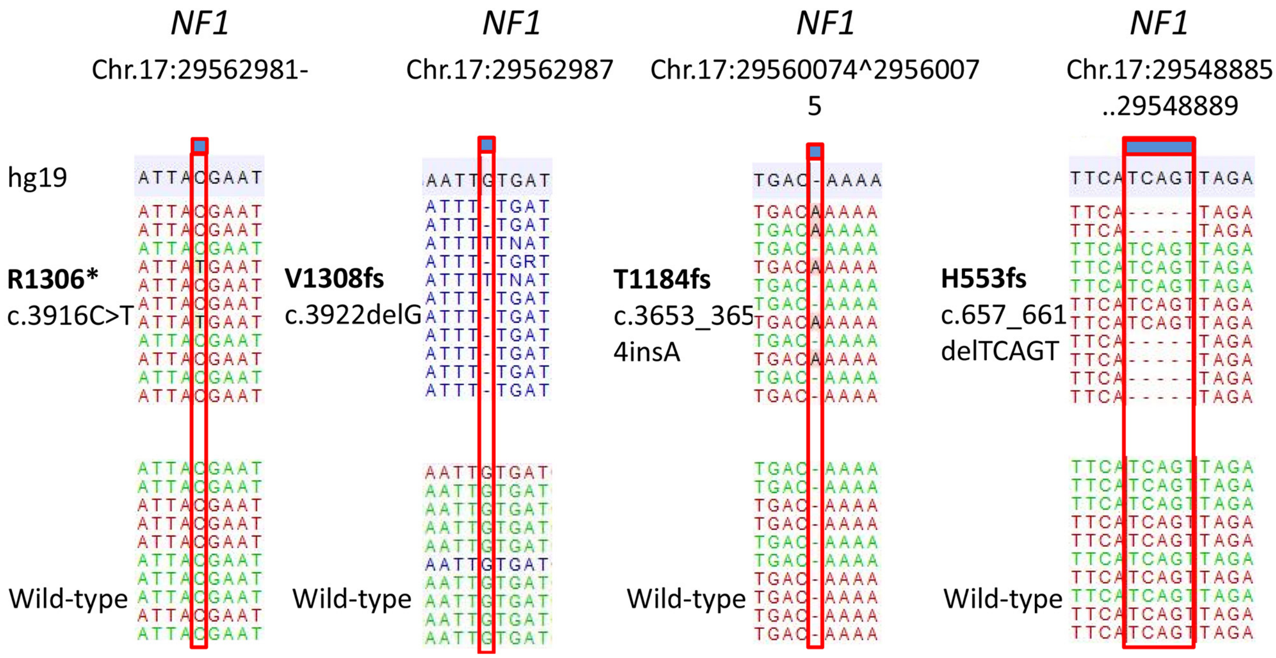

Supplementary Figure 1: Inactivating *NF1* mutations in mucosal melanomas. Targeted sequencing results of four exemplary mucosal melanoma samples revealing inactivating *NF1* mutations. Mutations are shown at the top with a representative wild-type sequence at the bottom. The mutation site is highlighted by the red box. Mutations are annotated according to human genome assembly 19 (hg19).

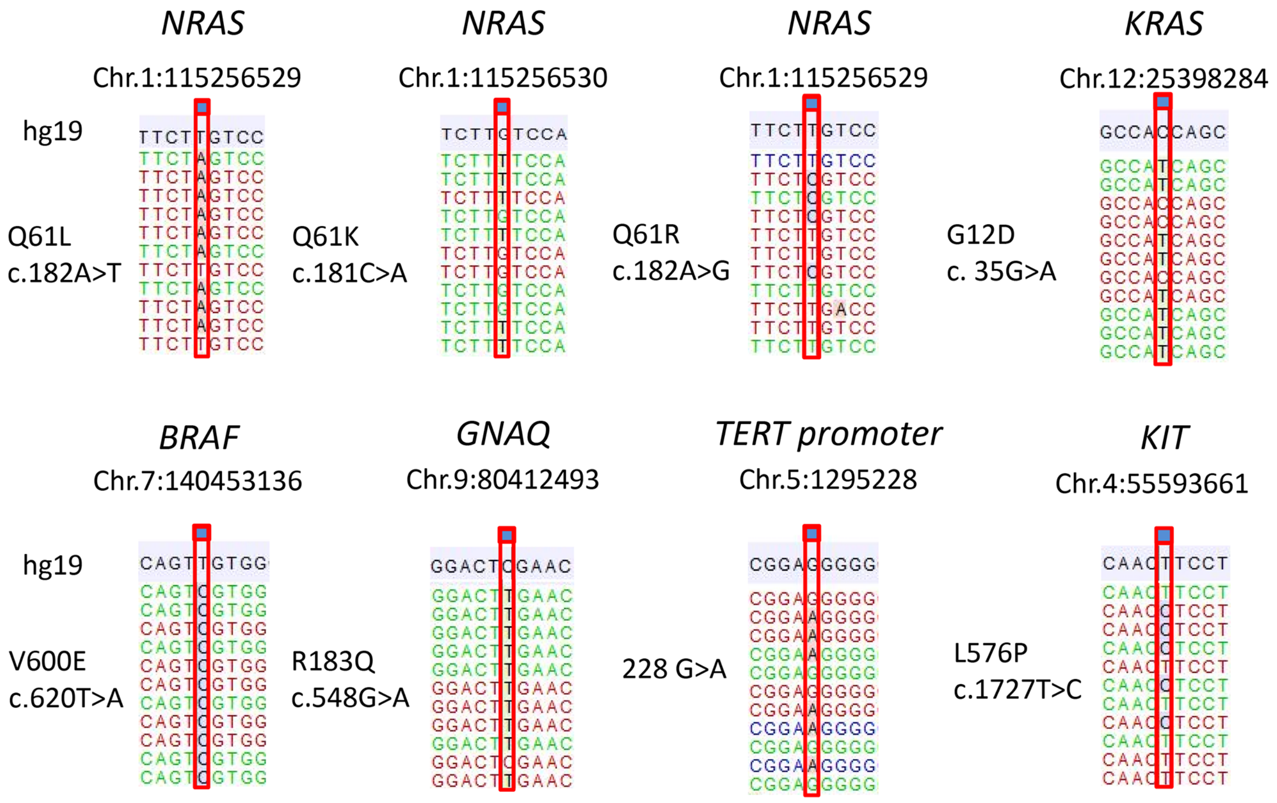

Supplementary Figure 2: Examples of activating mutations in mucosal melanomas. From left to right, representative examples of *NRAS, KRAS, BRAF, GNAQ, TERT promoter* and *KIT* mutations determined by targeted next generation sequencing. The mutation site is highlighted by the red box. Mutations are annotated according to human genome assembly 19 (hg19).

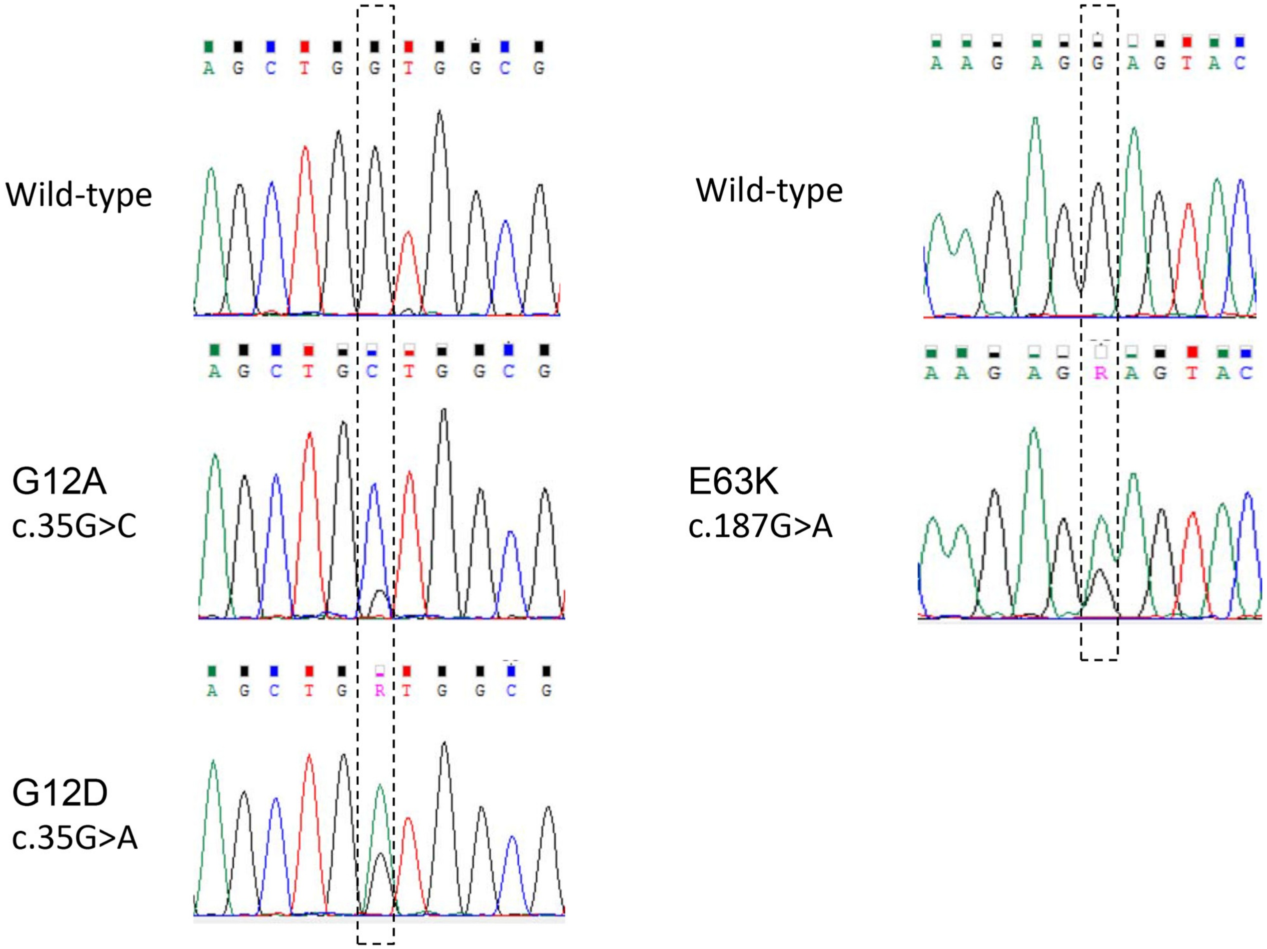

Supplementary Figure 3: Sanger sequencing of NGS identified *KRAS* mutations. Demonstrated are chromatograms of Sanger sequencing validating the *KRAS* mutations identified by our next-generation sequencing panel approach. Wild-type chromatograms are shown on the top. The G12A (c.35G>C) and G12D (c.35G>A) mutations identified in exon 1 are demonstrated on the left, the E63K (c.187G>A) mutation in exon 2 is shown on the right.

Supplementary Table 1: List of analyzed tumor samples with cDNA annotation and allele frequencies.

See Supplementary File 1

Supplementary Table 2: Clinical and pathological data of *BRAF*-mutant tumors

					Treatment		
***BRAF* mut.**	**site**	**sex**	**tissue**	**epithelial tumor component**	**BRAFi**	**BRAFi plus MEKi**	**alive**
V600E	HN	M	P	yes	no	no	no
V600E	G	F	P	yes	no	no	no
V600K	A	F	P	yes	yes (1 year)<br>Partial response*	no	no
V600E	HN	M	M	NK	no	no	no
V600E	D	F	P	yes	no	no	no
N188S	G	F	P	yes	no	no	no

M metastasis; *P* primary tumor; *NK* not known; *HN* Head and Neck, *G* Genital area, *A* Anorectum, *D* Digestive tract,
* patient initially showed a partial response then stable disease and upon progression after 1 year BRAF inhibitor therapy was discontinued.

Supplementary Table 3: Targeted next generation sequencing panel

Nr.	Gene	Primary Melanoma type	Customary Mutation Type	Target Bases	Bases covered	Primer pairs
1	BRAF	cutaneous	activating	2860	2456	40
2	NRAS	cutaneous	activating	650	650	10
3	KIT	cutaneous	activating	3354	3264	51
4	HRAS	cutaneous	activating	780	667	11
5	KRAS	cutaneous	activating	787	787	13
6	CDKN2A	cutaneous	tumorsuppressor	1184	713	14
7	PTEN	cutaneous	tumorsuppressor	1392	1248	22
8	CDK4	cutaneous		1052	1052	19
9	TP53	cutaneous	tumorsuppressor	1503	1396	26
10	RAC1	cutaneous		776	721	14
11	NF1	cutaneous	tumorsuppressor	9900	9167	143
12	PIK3CA	cutaneous		3607	3313	50
13	MAP2K2	cutaneous		1423	1240	24
14	PIK3R1	cutaneous		2637	2627	42
15	MITF	cutaneous		2066	2066	35
16	TERT	cutaneous		3719	2371	39
	TERT Promoter	cutaneous	activating	28	28	1
17	ARID2	cutaneous	tumorsuppressor	5928	5830	82
18	ARID1A	cutaneous	tumorsuppressor	7258	6132	81
19	SMARCA4	cutaneous	tumorsuppressor	5761	5040	88
20	MAP2K1	cutaneous		1436	1436	26
21	CTNNB1	cutaneous		2626	2626	40
22	EZH2	cutaneous		2680	2680	46
23	IDH1	cutaneous		1405	1394	22
24	FBXW7	cutaneous		2898	2808	43
25	WT1	cutaneous		1784	1282	24
26	GNAQ	uveal	activating	1220	1064	17
27	GNA11	uveal	activating	1220	944	14
28	BAP1	uveal	tumorsuppressor	2599	2380	39
29	SF3B1	uveal		4455	4412	72
