# Supplementary material for: Targeted next generation sequencing of mucosal melanomas identifies frequent NF1 and RAS mutations
Source: Oncotarget. 2017 Mar 24;8(25):40683–92. doi: 10.18632/oncotarget.16542 (PMC5522195; doi:10.18632/oncotarget.16542)
Supplement: Supplementary file 2 [file oncotarget-08-40683-s002.docx]

**Supplemental Table 1. List of analyzed tumor samples with cDNA annotation and allele frequencies**

| Nr. | Type | Location of primary | Tumor percentage % | NF1 | RAS | BRAF | Other Mutations |
| --- | --- | --- | --- | --- | --- | --- | --- |
| 1 | M | G | 90 | E2174fs, c.6577_6578delGA (43.1);L151fs, c.3975-8_3979delTTTTGTAGGTTAG (40.7) |  |  |  |
| 2 | M | DM | NK | R106*, c.1318C>T(48.5) |  |  |  |
| 3 | M | G | NK | R2258*, c.6772C>T (81.7) | KRAS G12D, c.35G>A (60.3) |  |  |
| 4 | P | A | NK | V1308fs, c.3922delG (10.5) |  |  | TERT S663N, c.1988G>A (11.5) |
| 5 | P | G | 100 | G1425fs, c.4274_4275insC (57.1) |  | V600E, c.620T>A (10.6) | TP53 Q165*, c.493C>T (69.6) |
| 6 | P | HN | NK | H553fs, c.1659_1663delTCAGT (44.2) |  |  | PIK3CA E109del, c.325_327delGAA (27.5) |
| 7 | P | G | NK | T889fs, c.2664_2665insA (30.8) |  | N188S, c.563A>G (23.0) |  |
| 8 | M | A | NK | R1306*, c.3916C>T (18.7) |  |  |  |
| 9 | P | HN | NK | T1184fs, c.3551_3552insA (46.9); D896N, c.2686G>A (43.5) | KRAS G12A, c.35G>C (86) |  | ARID1A V700A, c.2099T>C (60.8); SF3B1 D894N, c.2680G>A (47.6) |
| 10 | M | G | NK | H55R, c.1166A>G (40.1) |  |  |  |
| 11 | P | A | NK | I183N, c.548T>A (42.1) |  |  | PTEN K163fs, c.486_487insA 79.1(); SF3B1 R625H, c.1874G>A (25.4) |
| 12 | P | D | NK | M1376V, c.4126A>G (27.2) |  | V600E, c.620T>A (14.4) | ARID1A R1202Q, c.3605G>A (87.7) ; ARID2 T1208A, c.3622A>G (42.8); MITF A401S, c.1201G>T (41.6) |
| 13 | P | HN | NK | V1308L, c.3922G>T (45.8) | KRAS E63K, c.187G>A (64.6) |  | SF3B1 R62H, c.1874G>A (61.6) |
| 14 | P | HN | NK |  | KRAS G12F, c.34_35delGGinsTT (23.2) |  |  |
| 15 | P | HN | NK |  | NRAS Q61R c.182A>G (16.8) |  |  |
| 16 | P | HN | NK |  | NRASQ61R c.182A>G (45.3) |  |  |
| 17 | P | HN | NK |  | NRASQ61K, c.181C>A (30.5) |  |  |
| 18 | P | HN | NK |  | NRAS G13R, c.37G>C (31.7) |  | TERT Ser1104Thr, c.3311G>C (22.2) |
| 19 | P | HN | NK |  | NRAS Q61K, c.181C>A (26.5) |  | TERT P C228T (80); RAC1 N92K, c.276T>A (20); GNA11 S267F, c.800C>T (22.8) |
| 20 | NK | G | NK |  | NRAS Q61L, c.182A>T (95.9) |  | SF3B1 V634A, c.1901T>C (51.7) |
| 21 | M | HN | 100 |  | NRAS A59D, c.176C>A (43.2) |  | TERT P C243T (93.1); TERT P C252T (93.9); SMARCA4 A152T, c.454G>A (13.6) |
| 22 | M | G | 60 |  | NRAS I46M, c.138A>G (28.2) |  |  |
| 23 | M | HN | NK |  |  | V600E c.620T>A (29.8) | TERT P C250T (60.8) |
| 24 | P | HN | NK |  |  | V600E c.620_621delTGinsAA (48.8) | PIK3CA L896fs, c.2688_2695delGTTTACAC (32.5) |
| 25 | P | A | NK |  |  | V600K c.619_620delGTinsAA (37.8) |  |
| 26 | M | HN | 80 |  |  |  | KIT L576P, c.1727T>C (61.5) |
| 27 | M | Ur | NK |  |  |  | TERT P C228T (61.4); WT1 D497N c.1489G>A (19.7) |
| 28 | M | G | NK |  |  |  | GNAQ R183Q c.548G>A (60.7); MITF V476I c.1459G>A (42.8) |
| 29 | P | G | 50 |  |  |  | TP53 P58fs c.173delC (24) |
| 30 | P | G | 80 |  |  |  | ARID2 Y612C c.1835A>G (41.8) |
| 31 | M | HN | NK |  |  |  | TERT L1002V c.3193C>G (75.4) |
| 32 | M | A | 80 |  |  |  | PTEN L108R, c.323T>G (55.9) |
| 33 | M | G | NK |  |  |  | PIK3R1 T239M c.716C>T (49.8) |
| 34 | P | G | NK |  |  |  | KIT Y553del c.1657_1659delTAT (70.4); MAP2K2 G286R c.856G>A (15.8) |
| 35 | P | G | NK |  |  |  | TERT R819H c.2456G>A (51.2) |
| 36 | P | HN | NK |  |  |  | MITF N267K c.801C>A (48.2) |
| 37 | P | A | NK |  |  |  | TP53 C135R c.403T>C (74.1) |
| 38 | R | HN | NK |  |  |  | KIT V50L c.148G>T (44); PTEN C136R c.406T>C (69.4) |
| 39 | P | G | 95 |  |  |  | TP53 P151A c.451C>G (26.9) |
| 40 | NK | G | NK |  |  |  | ARID2 M545I c.1635G>A (53.1); SF3B1 R625H c.1874G>A (28.9) |
| 41 | P | G | NK |  |  |  | TERT R819H c.2456G>A (37.3) |
| 42 | NK | A | NK |  |  |  | MITF V487I c.1400T>A (39.9); SMARCA4 R1260S c.3780A>T (46.3) |
| 43 | R | HN | NK |  |  |  | SF3B1 T916S c.2746A>T (45.2); CK4 R209C c.625C>T (55.6) |
| 44 | P | HN | NK |  |  |  | TP53 R175G c.523C>G (65.9) |
| 45 | P | G | 90 |  |  |  | KIT L783I c.2347C>A (57.1) |
| 46 | M | DM | 80 |  |  |  | KIT I748Y c.2243T>C (50.2); BAP1 G579R c.1735G>A (52.2); SF3B1 R625L c.1874G>T (12.9) |
| 47 | P | HN | NK |  |  |  | BAP1 Y646C c.1937A>G (23.2) |
| 48 | P | HN | NK |  |  |  | CTNNB1 Y331C c.992A>G (41.5) |
| 49 | P | HN | NK |  |  |  | TERT S953F c.2858C>T (23.1) |
| 50 | M | DM | 95 |  |  |  | CK4 V174M c.520G>A (61) |
| 51 | R | HN | NK |  |  |  |  |
| 52 | P | HN | 80 |  |  |  |  |
| 53 | M | G | NK |  |  |  |  |
| 54 | P | D | NK |  |  |  |  |
| 55 | NK | G | NK |  |  |  |  |
| 56 | P | HN | NK |  |  |  |  |
| 57 | M | Ur | 80 |  |  |  |  |
| 58 | P | G | 75 |  |  |  |  |
| 59 | M | D | NK |  |  |  |  |
| 60 | P | HN | NK |  |  |  |  |
| 61 | P | HN | 90 |  |  |  |  |
| 62 | P | A | 95 |  |  |  |  |
| 63 | P | G | NK |  |  |  |  |
| 64 | P | G | NK |  |  |  |  |
| 65 | M | G | 85 |  |  |  |  |
| 66 | NK | G | 100 |  |  |  |  |
| 67 | M | HN | 100 |  |  |  |  |
| 68 | M | Ur | 70 |  |  |  |  |
| 69 | P | HN | NK |  |  |  |  |
| 70 | P | HN | NK |  |  |  |  |
| 71 | P | A | NK |  |  |  |  |

Green – mutations known to be activating; red – loss of function mutations; black – missense mutation (with unknown functional consequences); *M* metastasis; *P* primary tumor; *R recurrence*; *NK* not known; *fs* frame shift; * = stop codon (nonsense mutation); *HN* Head and Neck, *G* Genital area, *A* Anorectum, *D* Digestive tract, *Ur* Urinary tract, *DM* data missing.

Allele frequencies are state in brackets after the cDNA annotation.
